# Enhancing Pavement Durability: Comparative Rheological Evaluation of Conventional and Rejuvenated Reclaimed Binders under Aging Conditions

**DOI:** 10.3390/ma17133305

**Published:** 2024-07-04

**Authors:** Asmasadat Dabiri, Hugo M. R. D. Silva, Joel R. M. Oliveira

**Affiliations:** Institute for Sustainability and Innovation in Structural Engineering (ISISE), Department of Civil Engineering, Advanced Production & Intelligent Systems Associated Laboratory (ARISE), University of Minho, 4800-058 Guimaraes, Portugal; id9822@alunos.uminho.pt (A.D.); joliveira@civil.uminho.pt (J.R.M.O.)

**Keywords:** reclaimed asphalt, rejuvenator, asphalt binders, short-term aging, long-term aging, rheological properties

## Abstract

A drawback of recycled mixtures containing reclaimed asphalt is their increased stiffness, further worsened by the accelerated aging of binders in extreme weather conditions. Previous studies have shown that while rejuvenating agents can mitigate some of these issues by improving flexibility and reducing brittleness, they often present challenges, such as performance variability and the potential for rutting. This study aims to develop an optimal blend of reclaimed bitumen, a rejuvenating agent, and pure bitumen to achieve rheological properties similar to a control 35/50 pen-grade bitumen for road paving. Hence, the rejuvenated binders comprised 30:70 blends of reclaimed asphalt bitumen and 50/70 pen-grade bitumen, adding 0.2% to 0.6% of a rejuvenating agent by mass of the reclaimed asphalt. Sample testing included conventional penetration grade, softening point, and viscosity tests, followed by dynamic shear rheometer tests under unaged, short-term, and long-term aging conditions. The results show that the binder blend with 0.4% rejuvenator closely resembles the rheological properties of 35/50 pen-grade bitumen. This blend exhibits a 20% to 55% stiffness reduction for recycled mixtures with 30% reclaimed asphalt. Notably, the rejuvenated binders exhibited a similar level of aging resistance to the control bitumen, with a marginal difference of less than 5% in aging ratios. Meanwhile, large strain amplitude tests showed the importance of defining maximum rejuvenating incorporation rates in recycled mixtures to avoid rutting problems, where binders with 0.4% rejuvenator doubled the rutting potential (J_nr_ values). This innovative study highlights the potential for enhancing recycled mixtures’ performance by evaluating rejuvenated reclaimed binders’ rheology subjected to different aging conditions, thus contributing to sustainability in pavement construction.

## 1. Introduction

Asphalt pavements are subjected to various environmental conditions and traffic loads throughout their life cycle. These factors, combined with the oxidation of bitumen in the pavement, cause deterioration over time, such as rutting at high temperatures, fatigue cracks at moderate temperatures, and low-temperature cracking [1,2,3]. While periodic repair and maintenance can extend the pavement’s lifespan, road quality decreases after a certain operational period, reducing its International Roughness Index. Thus, asphalt surface milling or pavement rehabilitation becomes inevitable. Each year, millions of tons of milled asphalt, known as Reclaimed Asphalt (RA), are produced worldwide [4,5]. According to the National Asphalt Pavement Association (NAPA), over 82 million tons of RA were produced in 2018 alone, underscoring the scale of this issue [5,6]. The most recent NAPA report [7], which covers data up to 2021, highlights that the industry reuses up to 95% of RA on new pavements. In 2021, approximately 94.6 million tons of RA were used in asphalt production, a considerable increase from previous years. According to the European Asphalt Pavement Association [8], 16 out of 33 countries declared approximately 46 million tons of RA available for reuse or recycling, with only a tiny amount being sent to landfills.

Reclaimed asphalt from pavement milling contains significant mineral and fossil resources, comprising approximately 95% aggregate materials and 5% bitumen. These resources are produced through exploration, extraction, production, and transportation, incurring substantial costs. This process consumes large amounts of non-renewable energy. However, this energy can be saved by implementing pavement recycling technologies or other circular economy approaches [9,10].

Therefore, road owners and paving contractors are motivated to include RA in pavement mix designs. This interest intensified in the early 1970s with the global rise in oil and fossil fuel prices [11,12]. International organizations, including the European Union, have emphasized using clean energy and reducing environmental pollutants [13,14]. As a result, various countries started incorporating reclaimed asphalt (RA) into their pavement rehabilitation plans. This approach offers significant benefits, such as reduced material preparation and transportation costs, lower fuel and energy consumption, decreased environmental pollutants, and better management of raw resources [11,15,16,17].

Over twenty European countries use RA in various asphalt mixtures, including hot, cold, and warm mixes, both on-site and in production plants [15]. The specific usage in a binder or surface course and the amount of RA in asphalt pavements vary by country [11,15,18]. However, reclaimed asphalt is usually recommended for binder courses, with a consumption rate between 15% and 30% [18,19,20,21]. Despite the advantages of using RA, its application in paving has faced challenges. The most critical issue is the significant increase in binder and mixture stiffness containing RA. This increase poses a heightened risk of pavement cracking caused by traffic loading and low-temperature cracking [22]. This stiffness increase is caused by the bitumen aging in RA, which results from oxidation and UV reactions during the operational period and changes its chemical structure [23,24].

Bitumen comprises saturates, aromatics, resins, and asphaltenes (SARA). Saturates, aromatics, and resins are part of the maltene phase of bitumen, while asphaltenes, the polar component, are dispersed within this phase [25]. Asif et al. [26] found that aging significantly impacts bitumen’s rheological, chemical, and thermal properties. This situation changes SARA fractions, reducing the aromatic content and increasing the asphaltene content. Consequently, the bitumen exhibits increased elastic behavior and stiffness modulus [27,28,29]. Those studies also state that reclaimed bitumen in the final binder significantly increases viscosity, softening point, and complex modulus while decreasing the penetration grade compared to pure bitumen. This alteration leads to a greater tendency for elastic behavior in both the bitumen and asphalt mixture. This behavior enhances the performance of bitumen and asphalt mixtures with RA in the DSR test, particularly at high temperatures, low loading frequencies, and rutting tests. However, results from fatigue tests, fracture tests, and the thermal stress restrained specimen test on asphalt mixtures containing RA, along with the bending beam rheometer and linear amplitude sweep tests on binders containing reclaimed bitumen, show a significant increase in cracking potential [30,31,32,33,34].

One of the main advantages of using a rejuvenating agent is that it effectively reduces the stiffness of aged bitumen in RA [35]. Rejuvenating agents, rich in the maltene phase, balance the ratio of aromatics to asphaltenes in aged bitumen, compensating for deficiencies and restoring the bitumen’s lost characteristics and chemical structure [30,36,37,38]. Rejuvenating agents are classified into three main categories: plant oil, waste-driven oil, and refinery-based oil [1]. Each of these categories encompasses various subsets [1,30]. Some studies have examined the rheological characteristics and chemical structure of rejuvenated, reclaimed bitumen using DSR, Gel Permeation Chromatography (GPC), and SARA fractioning tests [1,39]. For example, SARA fractioning tests show how rejuvenating agents increase the maltene phase (especially non-polar aromatics) relative to asphaltenes by measuring the ratio and amounts of bitumen components. This change in chemical structure restores the primary properties of aged bitumen, enhancing its softness and flexibility [39]. Sun et al. [40] stated that rejuvenated binders with increased waste engine oil ratios facilitate diffusion toward virgin asphalt, strengthen molecular attraction, and promote the blending of virgin and aged binders.

The GPC test reveals changes in the molecular size distribution of bitumen components when a rejuvenator is used. According to Daly et al. [41], the components of the maltene phase have a molecular weight of less than 3000 Dalton, while the molecular weight of asphaltenes ranges from 3000 to 9000 Da. GPC analysis shows that, compared to virgin bitumen, reclaimed bitumen has a significantly higher percentage of particles with molecular weights greater than 3000 Da. Conversely, reclaimed bitumen containing a rejuvenating agent has a reduced rate of particles with molecular weights above 3000 Da, given the increased maltene-to-asphaltene ratio [17,41].

The Dynamic Shear Rheometer (DSR) test results on reclaimed bitumen with a rejuvenating agent also showed the benefit of increasing the bitumen’s viscous parameter (i.e., loss modulus, G″) at low temperatures and high loading frequencies. Therefore, the rejuvenator effectively improves bitumen performance [17,31,33,42,43]. However, the effectiveness of the rejuvenating agent in restoring the characteristics of reclaimed bitumen and aligning its viscoelastic behavior with that of pure bitumen depends on several factors. These include the type of rejuvenating agent, the amount used (i.e., typically ranging from 0.2% to 0.4%), and the method of adding rejuvenators to the mixture of virgin and reclaimed bitumen [30,36,37,38].

Cavalli et al. [30] conducted extensive tests on rejuvenated reclaimed bitumen using three bio-based rejuvenators at a rate of 5% of the pure bitumen’s weight. The results showed that cashew nutshell oil and tall oil, compared to natural seed oil, were more effective in restoring the rheological behavior of reclaimed bitumen under different temperatures and loading conditions. However, the best-performing rejuvenator (i.e., cashew nutshell oil) could not completely restore the reclaimed binder’s behavior to mimic pure bitumen, showing that the effectiveness of the rejuvenator depends on its type. Ma et al. [17] investigated the rheological behavior of virgin bitumen incorporated with 20% reclaimed bitumen and varying percentages of rejuvenators using DSR and GPC tests. These authors found that directly adding a rejuvenator to reclaimed bitumen yielded better results than adding it to virgin bitumen. Various other studies have explored different rejuvenators, including waste cooking oil (WCO), waste engine oil (WEO), tung oil, tall oil, Mahua oil, and vegetable oil [23,30,31,36,44,45,46], as well as other self-developed compound rejuvenators [47].

While the previous studies showed improved behavior of rejuvenated reclaimed bitumen against cracking, some drawbacks and challenges remained at high temperatures owing to bitumen’s tendency towards a more viscous behavior after rejuvenation [48]. Thus, some additional works [49,50,51,52,53] mentioned that the use of inappropriate types or quantities of rejuvenators (i.e., ordinary rejuvenators, excessive use) could soften the reclaimed asphalt too much and lead to reduced rutting performance (i.e., augmented non-recovered deformation) due to increased phase angle and decreased stiffness at higher temperatures.

The critical aim of using RA in asphalt pavement is to maximize its environmental and cost-related benefits while maintaining the same technical characteristics as pavements produced with pure bitumen and virgin materials. The primary challenge lies in the differences between the rheological properties of pure and RA’s aged bitumen. Achieving this balance requires selecting an appropriate combination of pure bitumen, reclaimed binder, and the type and amount of rejuvenating agent. Considering the present goals of the circular economy in Portugal for reducing waste disposal by increasing reuse and recycling and the recommended RA consumption rates for binder courses [18,19,20], a road owner and a paving contractor defined the challenge of incorporating 30% reclaimed asphalt as a standard practice in highway rehabilitation.

This study aims to respond to that challenge by assessing the potential for enhancing the recycled mixtures’ performance by evaluating rejuvenated reclaimed binders’ rheology subjected to different aging conditions. Thus, 30% reclaimed binder was blended with pure 50/70 pen-grade bitumen and various amounts of a rejuvenator or an alternative regenerator binder. The rheological characteristics of the bitumen samples produced with these components were investigated and compared to those of 35/50 pen-grade bitumen.

Provided that limited methodologies are available for addressing the aging resistance of rejuvenated binders, one of the innovative contributions of this study is the thorough rheological evaluation of the effects of short- and long-term aging on rejuvenated reclaimed bitumen compared to pure bitumen.

## 2. Materials and Methods

**Figure 1:**
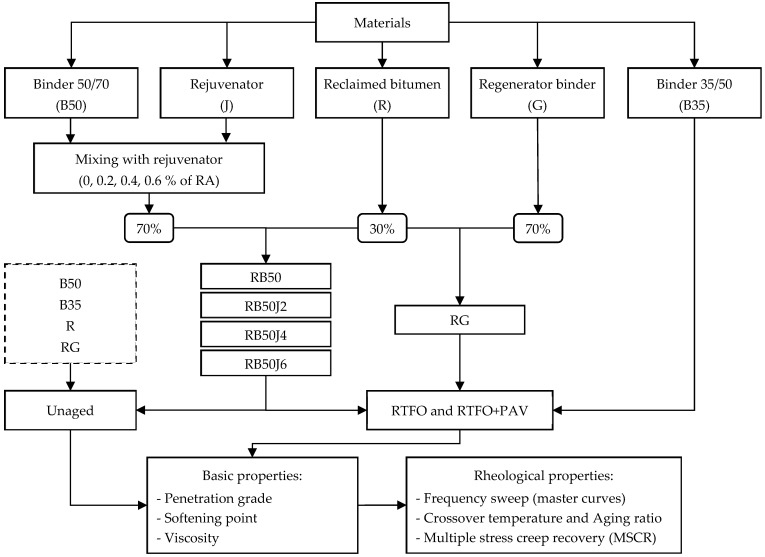
Experimental research flowchart.

The laboratory process in this research aims to achieve a suitable combination of pure bitumen, rejuvenator, and bitumen recovered from RA with performance properties similar to pure 35/50 pen-grade bitumen under various temperatures and loading conditions. Therefore, the rheological behavior of pure and reclaimed bitumen containing different amounts of the rejuvenating agent was assessed under three conditions: unaged, short-term, and long-term aged. The flowchart in Figure 1 outlines the details of the laboratory process and provides abbreviations for different bitumen samples.

### 2.1. Materials

The RA used to produce the recycled asphalt mixtures was provided by the Socorpena company from their asphalt plant in Vila Pouca de Aguiar, Portugal. Particle size distribution was assessed according to EN 12697-2 [54], and the results are presented in Table 1. Then, the amount of bitumen in the RA material was determined to be 5.5%, following the EN 12697-39 standard [55].

A pure 35/50 pen-grade bitumen, known for its relatively hard consistency and widespread use in the production of asphalt mixtures in Portugal, was sourced. In addition, a pure 50/70 pen-grade bitumen, characterized by its softer consistency, was requested for blending with the reclaimed binder. Finally, a commercial regenerator binder with a specific formula (Regener R80) was employed for mixing with the reclaimed binder. The Cepsa Company in Portugal provided all new binders.

A bio-based rejuvenator (Iterlene ACF 2000 Green, supplied by Iterchimica, Suisio, Italy), which is a mixture of vegetal derivatives, was used in quantities of 0.2%, 0.4%, and 0.6% by weight of RA (i.e., dosage range suggested by the supplier) as an additive to enhance the technical characteristics of the reclaimed binder when blended with pure 50/70 bitumen. The specifications of the rejuvenator used are detailed in Table 2.

### 2.2. Sample Preparation

#### 2.2.1. Preparation of the Reclaimed Binder

**Figure 2:**
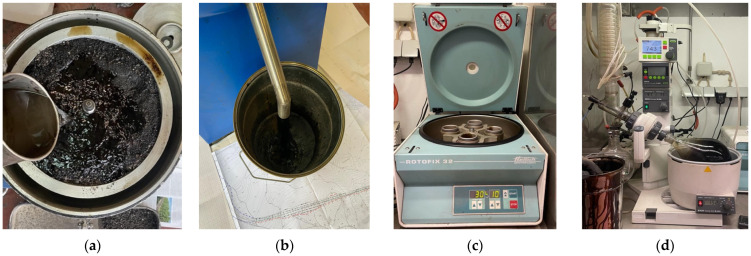
RA bitumen recovery process: (**a**) placing and submerging in the centrifuge; (**b**) extracting bitumen and toluene; (**c**) separation of fine material from the liquid phase; (**d**) distillation process.

The bitumen recovery process followed the method described in EN 12697-3 standard [56]. Approximately 6 kg of RA material was placed in a Rotarex centrifuge extractor submerged in one liter of solvent (toluene) for 30 min. The centrifuge underwent five consecutive cycles to separate bitumen and toluene from the RA. After each cycle, the bitumen and toluene returned to the centrifuge, starting a new extraction cycle. Subsequently, using a different centrifuge (Hettich Rotofix 32, Andreas Hettich GmbH & Co. KG, Tuttlingen, Germany), operating at 3000 rpm, fine material was separated from the base phase (bitumen and toluene). Finally, bitumen was separated from the toluene solvent through the distillation process in the Buchi rotavapor R-300, rendering it ready for further testing. Figure 2 illustrates the steps involved in recovering bitumen from RA.

#### 2.2.2. Preparation of the Rejuvenated Binder

Rejuvenated bitumen samples were prepared using two different binders. First, 30% of the reclaimed binder (R) extracted from the RA material was blended with 70% of soft 50/70 pen-grade bitumen, along with the rejuvenating agent (0.2%, 0.4%, and 0.6% by mass of the RA material, equivalent to 3.6%, 7.3%, and 10.9% of the mass of reclaimed bitumen, respectively).

For the second binder (RG), 70% of the commercial regenerator binder Regener R80 (G), with specific properties, was mixed with 30% of the reclaimed binder (R).

For each binder, a single blend was prepared at a temperature of 160 °C using an IKA RW20 DZM mixer operating at 200 rpm for 5 min to avoid creating additional variability and ensure consistency at this stage. However, the binder was enough to prepare the samples needed for aging conditioning and further testing, ensuring the repetitions specified for each test in Section 2.3.

#### 2.2.3. Preparation of the Short- and Long-Term Aged Binders

The rejuvenated reclaimed binder and pure bitumen samples underwent aging conditioning in two stages. First, the conditioning in the Rolling Thin Film Oven (RTFO) apparatus simulates short-term aging according to EN 12607-1 standard [57]. Following the previous steps, the unaged binder samples were exposed to short-term aging at 163 °C for 85 min.

Second, the long-term aging conditioning used the Pressure Aging Vessel (PAV) apparatus at 100 °C and 2.1 Mpa air pressure for 20 h on the RTFO samples, following EN 14769 standard [58].

### 2.3. Test Methods

#### 2.3.1. Physical Properties of Bitumen Samples by Conventional Tests

**Figure 3:**
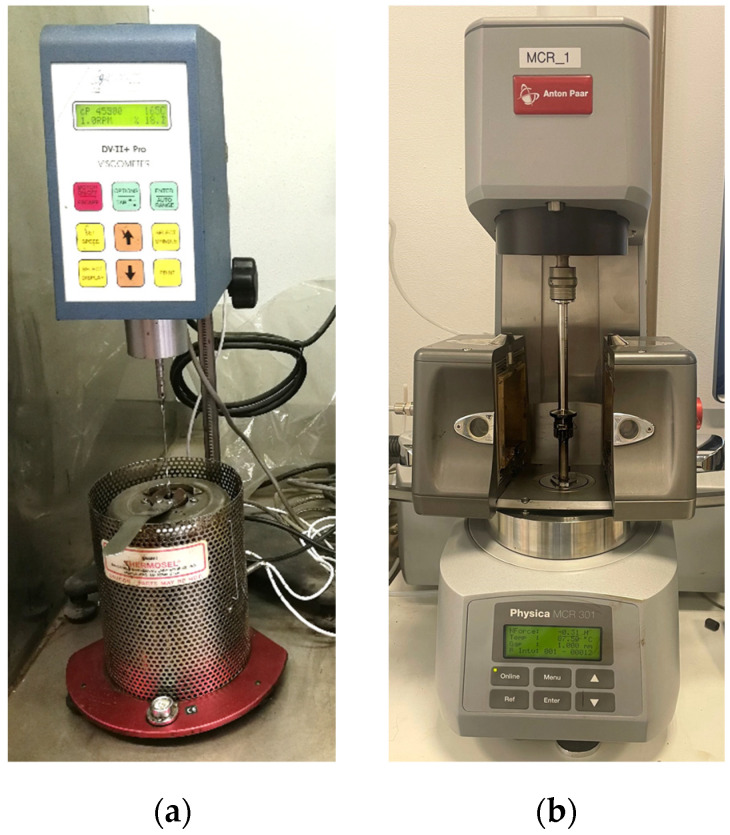
Examples of testing equipment used in this work: (**a**) rotational viscometer (Brookfield DV-II+Pro); (**b**) dynamic shear rheometer (Anton Paar Physica MCR 301).

Initially, conventional tests were conducted to determine the fundamental physical properties of pure, reclaimed, and rejuvenated bitumen samples under unaged and different aging conditions. Those tests included penetration grade with a needle at 25 °C, using a standard digital penetrometer according to EN 1426 [59], a softening point with the ring and ball apparatus according to EN 1427 [60], and dynamic viscosity at different temperatures (ranging from 120 to 180 °C) with a Brookfield rotational viscometer DV-II+Pro following EN 13302 standard [61] (Figure 3a).

The standards mentioned that the test results correspond to the mean value of three test repetitions for penetration and duplicate tests to evaluate the softening point and dynamic viscosity. All tests conducted in this study adhered to the following precision limits defined in the relevant standards:

- An absolute error of less than 2 for penetration values below 50 × 0.1 mm or a percentage error of less than 4% for values above this penetration threshold.
- An absolute error of less than 1 for the softening point test.
- A percentage error of less than 3.5% for the dynamic viscosity test.

#### 2.3.2. Complex Modulus and Phase Angle Master Curves under Small Strain Amplitude Rheological Tests

Dynamic Shear Rheometer frequency sweep tests were performed in an Anton Paar Physica MCR 301 rheometer (Figure 3b) at twelve different frequencies (ranging from 0.1 to 15.9 Hz) and sixteen different temperatures (ranging from 0 to 88 °C), according to the European standard EN 14770 [62], to calculate the rheological parameters of the binders mentioned above. This range of temperatures also adequately represents the Portuguese climatic region, an Atlantic coastal country with mild temperatures and very few records of negative values. These rheological tests were performed under small and controlled strain amplitudes to assess the binder properties in the linear region of their viscoelastic behavior. Therefore, a plate with a diameter of 8 mm and a strain level of 0.05% was used in the temperature range of 0 to 40 °C, while a plate with a diameter of 25 mm and a strain level of 0.1% was used in the temperature range of 46 to 88 °C.

Bitumen is a viscoelastic material, meaning the contribution of elastic and viscous components to its behavior can vary under different temperatures and loading conditions. The complex shear modulus (G*) serves as an indicator of bitumen’s resistance to deformation. G* comprises two components: the storage modulus (G′), representing the elastic parameter, and the loss modulus (G″), representing the viscous parameter. Both parameters vary as a function of loading and temperature. G′ reflects the energy storage capacity in bitumen during deformation, while G″ represents the energy dissipation. The phase angle (δ) evaluates the relative relationship between the two components G′ and G″ and measures the material’s response time to applied stress. The phase angle value ranges from δ = 0° for fully elastic behavior to δ = 90° for fully viscous behavior [18,43].

This study explored the rheological behavior of the different bitumen samples under unaged and aged conditions using a dynamic shear rheometer at varying temperatures and frequencies, with corresponding measurements of the viscoelastic components. A master curve was generated employing the time–temperature superposition principle for a reference temperature of 40 °C, as suggested by Yu et al. [18], and RheoCompass version 1.32.258 software specifically designed for use with Anton Paar rheometers. The equivalent frequency of each temperature in the experimental test was determined using the shift factor and Williams–Landel–Ferry (WLF) relations (Equations (1) and (2)).


(1) $$\log a_{T}=\frac{-C_{1}\left(T-T_{\mathrm{ref}}\right)}{C_{2}+\left(T-T_{\mathrm{ref}}\right)}$$

(2) $$a_{T}=\frac{F_{\mathrm{ref}}}{F}$$

In which:

a_T_: shift factor,

T: test temperature,

T_ref_: reference temperature (40 °C in this research),

F_ref_: equivalent frequency at the reference temperature,

F: frequency at the desired temperature,

C_1_ and C_2_: Constant coefficients.

The master curve results presented in this work are the best-fit models of two DSR test repetitions at each temperature and frequency, using a Fourier log-log fit for complex modulus and a Gaussian semi-log fit for the phase angle. All adjustments were conducted in Matlab R2023a version 9.14.0.2286388 software, and the high coefficients of determination (R^2^ > 0.98) demonstrate the model’s fitting accuracy. A few outliers (i.e., measurements affected by machine compliance or outside the range of the test geometry for specific temperatures and frequencies) were eliminated. In addition, individual measurements were validated by the acceptability criteria mentioned in the relevant standard for the complex modulus (percentage error lower than 15%) and phase angle (absolute error lower than 3°).

#### 2.3.3. Crossover Temperatures under Small Strain Amplitude Rheological Tests

In this research, to better understand the viscoelastic behavior of bitumen, the changes in the storage modulus (G′) and loss modulus (G″)—elastic and viscous components of the complex modulus—at three frequencies: 0.1 Hz (low frequency or high temperature), 1.59 Hz (middle frequency), and 10 Hz (high frequency or low temperature) were investigated. At low temperatures, bitumen behaves elastically, so in this range, the value of G′ is higher than G″. However, as the temperature rises, bitumen undergoes a transition to a more viscous state. Then, at a certain point, G″ becomes higher than G′. The intersection of the G″ and G′ curves at a specific temperature signifies the crossover temperature of the elastic and viscous bitumen components [43,63]. Crossover temperature is a significant parameter, as it delineates the transition between the elastic behavior domain (controlled by G′) and viscous behavior (controlled by G″) in bitumen.

In this work, the crossover temperatures were calculated from mean DSR test results conducted in duplicate. The percentage errors for G′ and G″ were consistently lower than 3%.

Understanding the crossover temperature and its change because of aging is crucial for assessing the rheological properties of bitumen and predicting its performance in asphalt mixtures. Below this temperature, the elastic response predominates, and bitumen is more resistant to deformation and capable of providing structural support to the pavement. Conversely, the viscous behavior dominates above the crossover temperature, and bitumen becomes more susceptible to flow and deformation under traffic loads [43,64]. Therefore, the crossover temperature significantly influences asphalt pavements’ mechanical properties and durability. A lower crossover temperature implies higher stiffness and better resistance to rutting, while a higher crossover temperature suggests improved flexibility and fatigue resistance. Thus, selecting bitumen with an appropriate crossover temperature is essential for designing asphalt mixtures that can withstand various environmental and traffic conditions while maintaining long-term performance [63].

#### 2.3.4. Aging Ratio Assessment under Small Strain Amplitude Rheological Tests

The aging effect on each bitumen sample depends on the DSR test frequency. Therefore, it is beneficial to compare the changes in the complex modulus between aged and unaged conditions over a wide range of frequencies to better understand the impact of aging on each bitumen sample. Thus, the Aging Ratio (AR) coefficient defined by Equation (3) can integrate the stiffness changes caused by bitumen aging over a wide range of frequencies.


(3) $$Aging\;Ratio=\frac{{\left[\int_{log\;EF\;min}^{log\;EF\;max}\left(G^{*}\right)d\left(log\;EF\right)\right]}_{aged}}{{\left[\int_{log\;EF\;min}^{log\;EF\;max}\left(G^{*}\right)d\left(log\;EF\right)\right]}_{unaged}}$$

The AR value represents the ratio between the area under the curve of aged bitumen and unaged bitumen in the log–log master curve of G* against equivalent frequency. This AR value considers a wide range of loading frequencies and temperatures to evaluate the aging effect on the rheological properties of bitumen samples, i.e., the increased stiffness of aged bitumen compared to unaged bitumen.

AR values were computed from the best-fit complex modulus master curves for two DSR test repetitions, as explained previously. This calculation was reasonable given the excellent model’s fitting accuracy (R^2^ higher than 0.98) for all binders under different aging conditions.

#### 2.3.5. Rutting and Fatigue Performance Parameters of Bitumen Samples

The parameter G*/sin(δ) is a criterion used to control rutting resistance. At a frequency of 1.59 Hz (10 rad·s^−1^), the value of this parameter should be at least 1000 Pa for unaged bitumen samples and 2200 Pa for samples subjected to short-term aging (RTFOT), as mentioned in Cominsky et al. [65].

Likewise, the rheological characteristics of asphalt binders related to their fatigue resistance can be evaluated at intermediate temperatures through changes in the G*·sin(δ) parameter. This parameter is measured in the 0 to 40 °C temperature range on bitumen samples aged by the PAV method.

Bitumen becomes more brittle and elastic as the temperature decreases, increasing the chance of cracking in bitumen and asphalt mixtures. According to Cominsky et al. [65], the maximum value of G*·sin(δ) for controlling fatigue cracking is 5000 kPa.

The rutting and fatigue performance parameters were also calculated from the duplicate DSR test mean. The corresponding percentage errors were consistently lower than 5% for G*/sin(δ) and 3% for G*·sin(δ).

#### 2.3.6. Large Amplitude Rheological Properties at High Temperatures

Large amplitude strain tests under stress control (multiple stress creep recovery or MSCR) were performed to evaluate the rutting resistance of the several binders before and after aging at the highest pavement in-service temperatures in Portugal (60 °C). These tests were conducted using the Physica MCR 301 rheometer (Anton Paar GmbH, Graz, Austria) according to the EN 16659 standard [66] to ensure that the type and amount of rejuvenator used would not cause rutting problems if the rejuvenated binder had excessive flow.

The test uses ten stress and recovery cycles to examine the percentage recovery (%R) and non-recovered deformation (J_nr_) of the bitumen samples. Each cycle involves applying stress for 1 s, followed by a 9-s recovery period. The bitumen samples are subjected to two stress levels (100 Pa and 3200 Pa) using a 25 mm plate with a 1 mm gap. However, the results will only be presented for the most demanding service conditions, i.e., the higher stress level of 3200 Pa.

The results of the MSCR tests presented in this study correspond to the mean values of two test repetitions. Those results adhered to the precision limits specified in the relevant standard: percentage errors of less than 1% for %R and 6% for J_nr_. All tests met these criteria, ensuring the reliability and accuracy of the findings.

## 3. Results and Discussion

### 3.1. Physical Properties of Bitumen Samples by Conventional Tests

**Figure 4:**
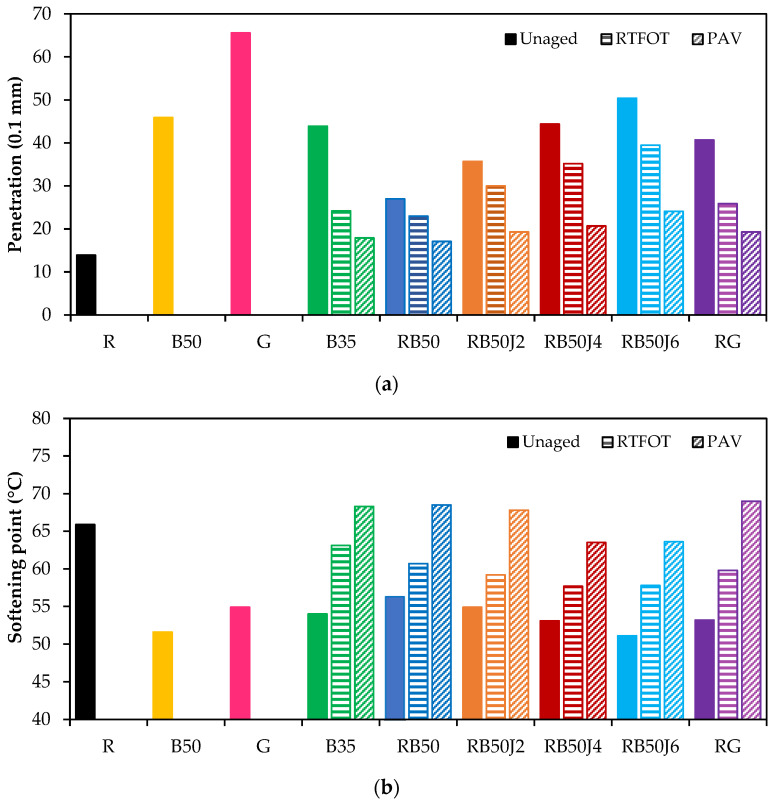
Basic characteristics of the different binders: (**a**) penetration; (**b**) softening point.

Figure 4 illustrates the changes in different bitumen penetration grades and softening points. The low penetration grade of reclaimed bitumen (R) confirms its high stiffness, resulting from long-term aging during the in-service period. In contrast, the R80 commercial binder (G) was the softest bitumen among the samples. Combining reclaimed bitumen R with soft bitumen B50 without rejuvenator (RB50) leads to an almost two-fold increase in the penetration grade. However, to achieve characteristics of the penetration grade similar to B35, using a rejuvenating agent with a minimum amount of approximately 0.4% is mandatory. A similar behavior is also explicit in the results of the softening point test.

The RB50J6 sample, incorporating 0.6% rejuvenator, exhibited the softest behavior compared to the other samples incorporating the rejuvenator and reclaimed binder. This percentage of rejuvenator consumption appears to be more than the amount required to meet the specifications of pure 35/50 pen-grade bitumen. Therefore, a behavior similar to that of 35/50 pen-grade bitumen, widely used in road paving, is expected within the range of 0.3% to 0.4% rejuvenator when added to a 70:30 blend of pure 50/70 pen-grade bitumen and reclaimed binder. Thus, thorough rheological characterization was only performed on the binders with 0.2% and 0.4% rejuvenator.

The effect of short- and long-term aging on rejuvenated reclaimed bitumen samples can also be analyzed in Figure 4. As expected, oxidation aging correlates with a decline in penetration values and a rise in softening point measurements in all samples.

Notably, aging in both short- and long-term conditions was more pronounced in the B35 sample than in the sample with a similar penetration grade and softening point (RB50J4). The decrease in penetration grade of the B35 sample under short- and long-term aging conditions was 45% and 60%, respectively, whereas these values were 20% and 52% for the RB50J4 sample. This lower decrease in the penetration grade of the rejuvenated binder should imply better in-service performance.

**Figure 5:**
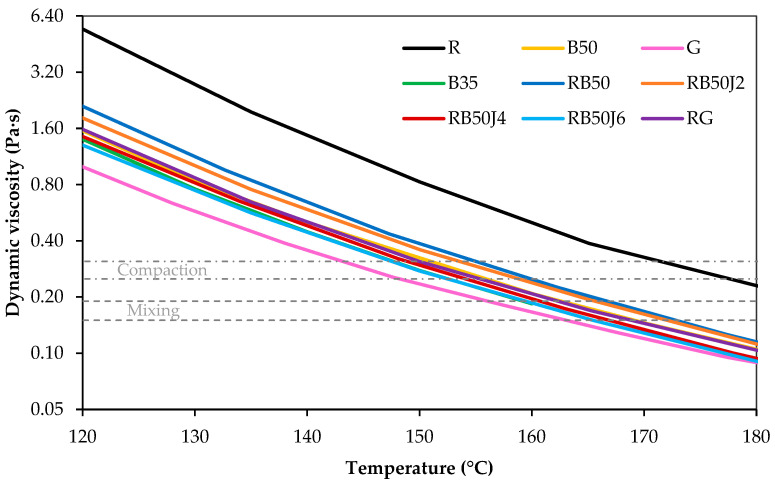
Dynamic viscosity test results of binders at different temperatures.

The viscosity values of the tested bitumen samples in the rotational viscometer at different temperatures are shown in Figure 5.

First, the remarkably high viscosity of the reclaimed bitumen (R) sample compared to other bitumen samples is notable. At a temperature of 135 °C, the viscosity of the reclaimed bitumen sample is almost two and a half times that of the pure 35/50 pen-grade bitumen (i.e., relatively hard bitumen), demonstrating the significant effect of aging on bitumen during the pavement service life. The commercial bitumen (G) is a very soft bitumen with the lowest viscosity. Blending it with the reclaimed bitumen significantly reduces its viscosity. A similar trend was observed in pure 50/70 pen-grade bitumen (B50) combined with reclaimed bitumen in a 70/30 ratio.

The utilization of a rejuvenating agent in this composition notably reduces the bitumen’s viscosity, particularly at higher usage percentages. This trend suggests that the rejuvenator restores the maltene phase of the reclaimed binder. The viscosity of the RB50RJ4 sample closely matches the B35 (35/50 bitumen) sample.

### 3.2. Small Strain Amplitude Rheological Properties of Unaged Bitumen Samples

#### 3.2.1. Complex Modulus and Phase Angle Master Curves

**Figure 6:**
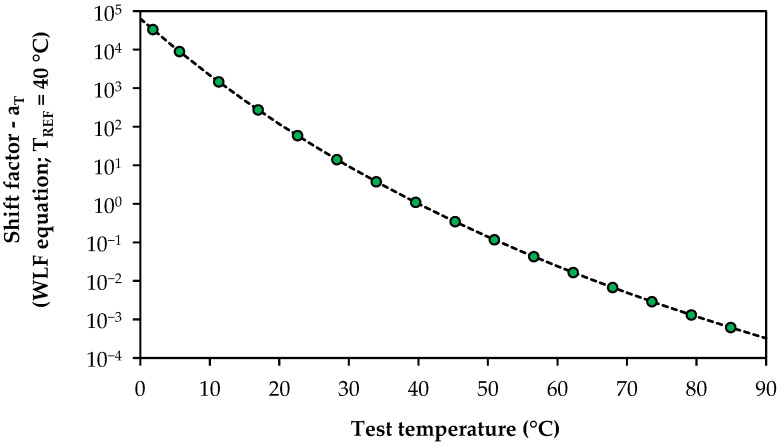
Example of the shift factor values used to plot the master curve of unaged bitumen B35 using the WLF equation and a reference temperature of 40 °C.

Different shift factor values were computed for each bitumen and test temperature using Equations (1) and (2), as exemplified in Figure 6 for the unaged B35 bitumen used as the control material in this work.

**Figure 7:**
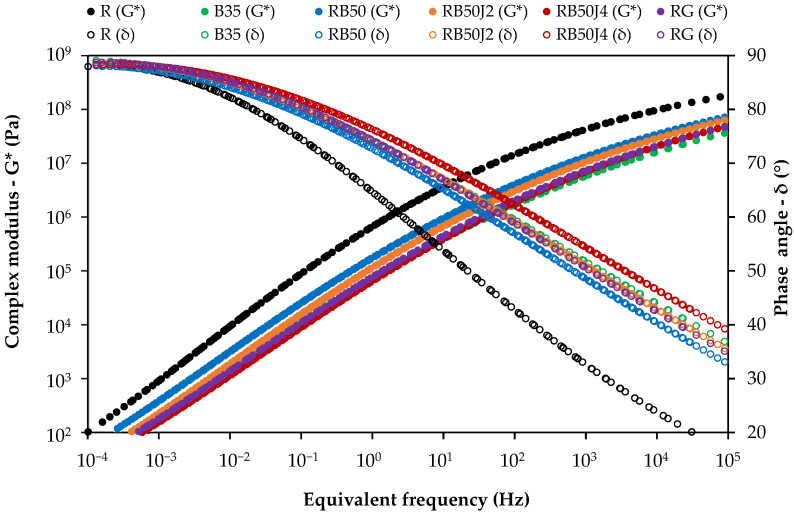
Complex modulus and phase angle master curves of unaged bitumen samples for a reference temperature of 40 °C.

The master curve diagram, illustrating the changes in the complex modulus and phase angle of the unaged bitumen samples before conducting the Rolling Thin Film Oven Test (RTFOT) and Pressure Aging Vessel (PAV) tests, is presented in Figure 7.

The graph illustrates that the reclaimed binder exhibits significantly higher complex modulus values and lower phase angle values across all temperatures and frequencies than other tested samples. This result confirms the bitumen’s high stiffness and elastic behavior, which is associated with the aging process occurring in bitumen after losing a significant portion of the maltene phase through oxidation during the service life. Combining the reclaimed binder with a softer bitumen and a rejuvenator decreases the complex modulus, reducing bitumen stiffness. However, these changes can vary across different frequency ranges.

At low frequencies (corresponding to higher temperatures) where bitumen inherently behaves viscously, the RB50 sample shows the highest complex modulus, surpassing pure 35/50 pen-grade bitumen. In contrast, bitumen samples containing the rejuvenator exhibit a lower complex modulus, with the lowest value observed in the RB50J4 sample (with an approximate stiffness reduction of 55% compared to the RB50 binder without a rejuvenator). Therefore, while combining reclaimed bitumen with softer bitumen (i.e., 50/70 pen-grade bitumen) significantly reduces stiffness, it remains higher than pure 35/50 pen-grade bitumen. However, all rejuvenated bitumen samples exhibited similar or lower stiffness than B35 bitumen.

This trend slightly differs at higher frequencies (corresponding to lower temperatures). The complex modulus of the RB50J4 sample closely matches that of the B35 and RG samples. In contrast, the RB50J2 samples exhibit the highest complex modulus, nearly equivalent to that without a rejuvenator. The phase angle results at high frequency also confirm this tendency, showing the highest delta value (i.e., softer behavior) in samples with 0.4% of the rejuvenating agent, highlighting the optimal performance of the agent at this concentration. Other samples that contain the rejuvenator display lower delta values and exhibit more elastic behavior than B35 bitumen.

In conclusion, while using a rejuvenating agent softens the bitumen at high frequencies (corresponding to low temperatures) where elastic behavior predominates, low percentages do not have a significant rheological effect on the rejuvenated bitumen. Likewise, combining 30:70 reclaimed bitumen and 50/70 bitumen without a rejuvenator fails to fully simulate the rheological behavior of 35/50 pen-grade bitumen because it presents lower phase angles.

This study found that rejuvenated binders containing 0.4% rejuvenator exhibited rheological properties similar to pure 35/50 pen-grade bitumen, reducing the complex modulus of RB50 binder by 55% (i.e., similar binder without rejuvenator). This observation aligns with studies by Cavalli et al. [30], who noted that certain bio-based rejuvenators effectively restored the rheological behavior of reclaimed bitumen under various temperatures and loading conditions. However, even the most effective rejuvenator could not fully match the properties of pure bitumen, emphasizing the challenge of replicating the virgin materials’ performance.

#### 3.2.2. Crossover Temperatures

**Figure 8:**
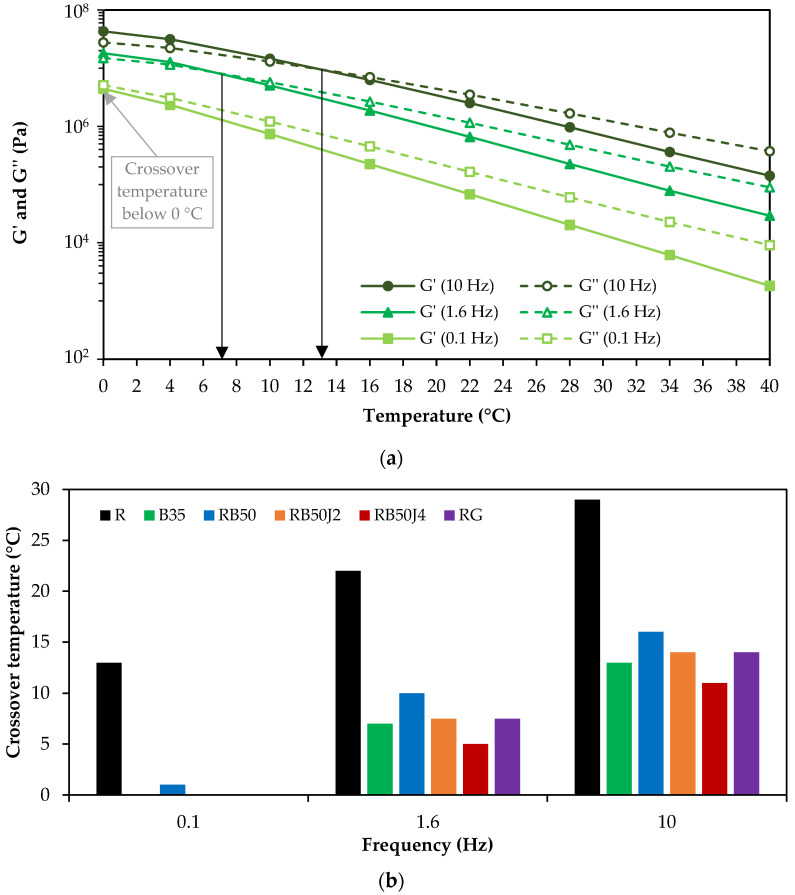
Unaged bitumen samples test results: (**a**) storage and loss modulus variation in B35 at different temperatures (the arrows show the crossover temperature); (**b**) crossover temperature of bitumen samples at different frequencies.

An example of the changes in viscoelastic components for sample B35 is shown in Figure 8a. At lower temperatures, the viscous component of the bitumen exceeds the elastic one, demonstrating softer behavior. Figure 8b displays the crossover temperature values for various bitumen samples at three different frequency levels.

The graphs in Figure 8a,b show that the crossover temperature in bitumen is lower at low frequencies. Hence, in all bitumen samples (except R and RB50), the crossover temperature is negative at 0.1 Hz (the G′ and G″ curves do not intersect at the test temperatures). With increased loading frequency, the crossover temperature rises in all samples, demonstrating increased elastic behavior in bitumen. The results show that the RB50 bitumen sample has the highest crossover temperature despite a significant reduction compared to the reclaimed binder. The lowest crossover temperature is observed in RB50J4, confirming the analysis performed in Figure 7.

The study assessed the storage modulus (G′) and loss modulus (G″) to understand the crossover temperature of the different binders. The results showed that rejuvenated binders had lower crossover temperatures than the corresponding binder RB50 without a rejuvenating agent, demonstrating increased viscous behavior at low temperatures and high loading frequencies. This observation aligns with studies by Cavalli et al. [30], who noted that rejuvenators could lower the crossover temperature, enhancing the viscous properties at low temperatures. Daly et al. [41] also highlighted the impact of rejuvenating agents on enhancing the maltene phase in aged bitumen, contributing to improved rheological behavior.

In summary, understanding the crossover temperature and the behavior of the viscoelastic components of bitumen under different conditions provides crucial insights into the material’s performance, especially when combining reclaimed bitumen with new materials and rejuvenators. This approach helps tailor the bitumen mixture to achieve the desired properties, ensuring optimal performance in various pavement applications.

### 3.3. Small Strain Amplitude Rheological Properties of Aged Bitumen Samples

#### 3.3.1. Complex Modulus and Phase Angle Master Curves

**Figure 9:**
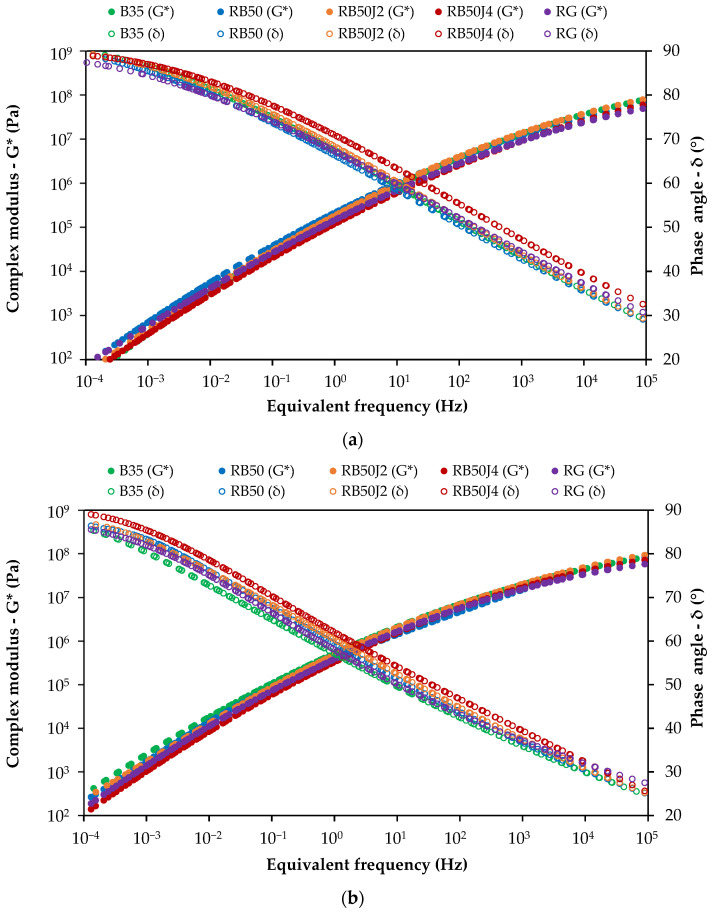
Complex modulus and phase angle master curves of the bitumen samples for a reference temperature of 40 °C: (**a**) short-term aging; (**b**) long-term aging.

Aging disrupts the ratio of maltene-to-asphaltene in bitumen. Although rejuvenators or softer bitumen can help recover the lost maltene in the reclaimed bitumen, it will still be subjected to additional aging if reused in pavements. Investigating the behavior of rejuvenated reclaimed bitumen under aging conditions is essential for understanding the long-term performance of rejuvenators and their ability to maintain the maltene-to-asphaltene ratio in bitumen. RTFO and PAV tests were conducted on the samples produced in this study to simulate short-term and long-term aging, respectively, and their rheological characteristics were evaluated using the DSR test. Figure 9a (RTFO) and Figure 9b (PAV) show the complex modulus (G*) and phase angle (δ) master curves after aging.

The master curves show that aging conditioning increased the complex modulus values and decreased the phase angle values for all tested bitumen samples, revealing increased stiffness and elastic behavior because of aging.

In short-term aging (RTFOT), the RG bitumen sample exhibited higher complex modulus values and lower phase angle values at lower frequencies than other samples, including pure bitumen B35, suggesting that short-term aging had a more significant effect on the RG sample than on pure bitumen. In contrast, the RB50J4 sample consistently showed the lowest complex modulus at low loading frequencies (high temperatures), highlighting the impact of the rejuvenator amount used in bitumen containing the reclaimed binder (with an average stiffness reduction of 35% compared to the binder without a rejuvenator, RB50).

At high loading frequencies, the stiffness of the bitumen samples follows almost the same pattern as the test results in unaged conditions. The RG and RB50J4 bitumen samples have the lowest complex modulus, while the RB50J2 sample has a higher complex modulus. Thus, it should be noted that short-term aging at high-loading frequencies has a more significant effect on B35. Changes in the phase angle also confirm this. The RG and RB50J4 samples exhibit a decreased elasticity at high frequencies, as evidenced by their highest δ values. At low frequencies (high temperature), higher complex modulus values (greater stiffness) are more desirable for preventing permanent deformations. In contrast, at high loading frequencies (low temperature), lower complex modulus values (lower stiffness) can help prevent cracking. Therefore, the RG sample performed well in short-term aging conditions in both cases.

Figure 9b shows the complex modulus and phase angle changes caused by long-term aging. Under the extreme conditions of the long-term aging test and the intensification of the oxidation process, the ratio of aromatics in the maltene phase decreases significantly compared to asphaltenes. As expected, there is an increase in complex modulus values and a decrease in the phase angle in all samples compared to the short-term aging. In contrast to the results observed in short-term aging, the highest complex modulus values at low loading frequencies belong to B35. Therefore, pure bitumen B35 has a higher aging potential in long-term aging conditions compared to the bitumen samples containing the rejuvenating agent.

In long-term aging conditions, the sample RB50J4 (with 0.4% rejuvenator) shows a lower complex modulus than the other bitumen samples. The stiffness of RB50J4 bitumen at high frequencies is still slightly below B35 bitumen. In addition, the average stiffness of this rejuvenated binder is 20% lower than the RB50 binder without a rejuvenator.

Similarly to the short-term aging condition, at high-frequency levels, the RG sample shows lower complex modulus values and the highest phase angle value, confirming the tendency of the bitumen to behave softer, which can be desirable at this frequency.

The impact of aging in both short-term and long-term conditions on different bitumen can vary depending on the type of bitumen and the type and amount of rejuvenating agent. In addition, this effect follows a distinct trend at different loading frequencies.

The innovative aspect of this study lies in its comprehensive evaluation of rejuvenated binders’ rheological properties under various aging conditions. The results show that the careful selection and proportioning of rejuvenators is essential to optimize the reclaimed binders’ performance depending on the expected pavement applications (e.g., RB50J4 behaved better under several conditions, but the RG sample stood out after long-term aging at high frequencies). This comparative approach is crucial for understanding how different rejuvenators impact the long-term performance of asphalt mixtures, as also suggested by other researchers in the field [18,30,40].

#### 3.3.2. Crossover Temperatures

**Figure 10:**
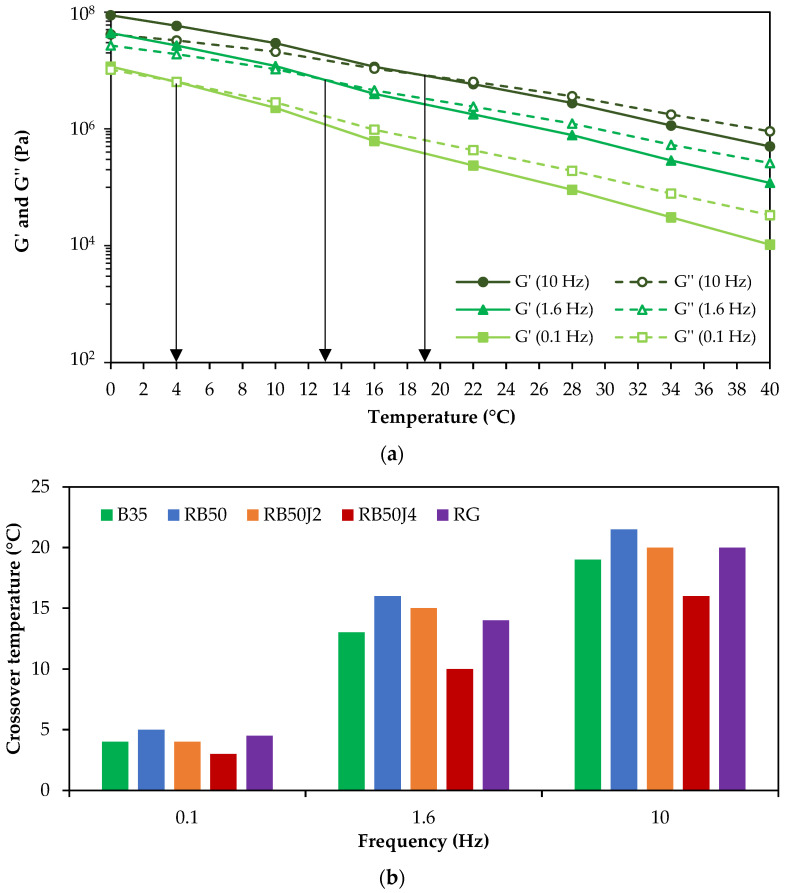
Short-term aged bitumen samples test results: (**a**) storage and loss modulus variation in B35 at different temperatures (the arrows show the crossover temperature); (**b**) crossover temperature at different frequencies.

**Figure 11:**
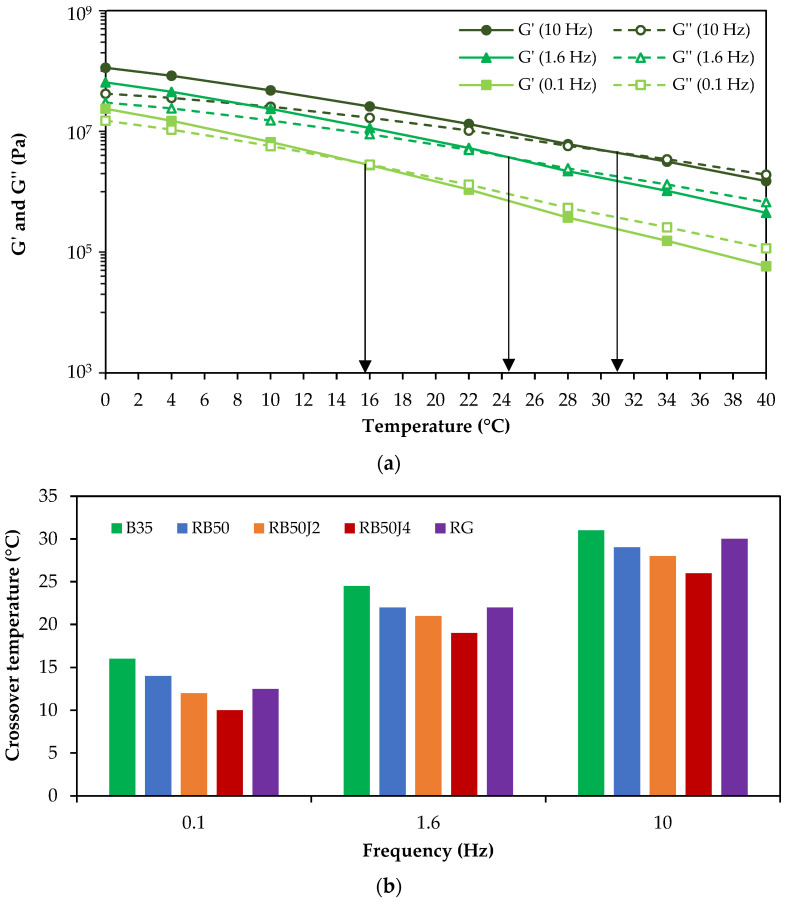
Long-term aged bitumen samples test results: (**a**) storage and loss modulus variation in B35 at different temperatures (the arrows show the crossover temperature); (**b**) crossover temperature at different frequencies.

Figure 10 (short-term aging) and Figure 11 (long-term aging) display the changes in the G′ and G″ of sample B35 under different temperature and aging conditions.

Compared to the unaged bitumen samples (Figure 8), the crossover temperature has increased because of the aging process and increased bitumen stiffness. This increase is more significant in long than short-term aging conditions.

The crossover temperature values of different bitumen samples after short-term aging at three frequency levels (low, medium, and high) are illustrated in Figure 10a,b. In all bitumen samples, the crossover temperature has increased with the rise in loading frequency. However, the trend of crossover temperature changes in short- and long-term aging conditions differs among the tested samples. RB50 bitumen has the highest crossover temperature in short-term aging conditions at high and low frequencies.

In contrast, in long-term aging conditions, B35 has the highest value, demonstrating the high effectiveness of the PAV test on aging the bitumen. The crossover temperature of bitumen RB50J4 shows the lowest value in both short- and long-term aging modes, demonstrating the lower stiffness of the binder with 0.4% rejuvenator compared to other samples (confirming the results shown in Figure 9a). These results also suggest that rejuvenated reclaimed binders are aging-resistant and can maintain a softer behavior after short- and long-term aging.

### 3.4. Aging Ratio Assessment under Small Strain Amplitude Rheological Tests

**Figure 12:**
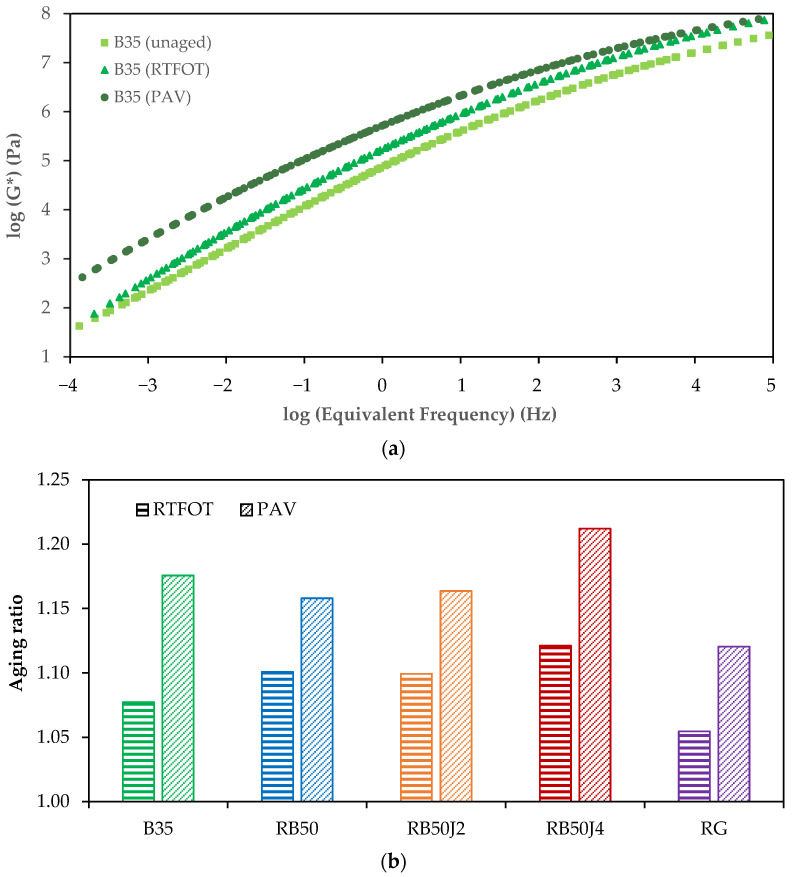
Aging ratio of bitumen samples: (**a**) example of the effect of aging conditions on the complex modulus of B35 binder; (**b**) comparison between the aging ratio of the different binders.

Figure 12a exemplifies the log (G*) changes against the log (Equivalent Frequency) for the control B35 bitumen.

As depicted in the graph, the curve of the complex modulus shifts to higher values after aging, showing an increase in bitumen stiffness. However, the trend of complex modulus changes differs between short-term and long-term aging conditions. The increase in complex modulus under short-term aging conditions is not significant at low frequencies, remaining close to the G* values of unaged bitumen. In contrast, the increase in the complex modulus is substantial at higher frequencies.

In long-term aging, an inverse trend is observed: the increase in complex modulus is more pronounced at low frequencies than at high frequencies. The effect of aging (i.e., RTFOT and PAV) on the rheological behavior of B35 bitumen varies across different frequencies. This trend may not apply to all tested bitumen samples.

Figure 12b shows the varying values of the aging ratio for the bitumen samples tested in this study. Despite RB50J4 exhibiting the lowest complex modulus value in the DSR test on the short-term and long-term aging samples, Figure 12b shows that RB50J4 has the highest AR value in both aging conditions. In contrast, the lowest AR value corresponds to bitumen RG. These results suggest that the rejuvenated bitumen’s performance against aging depends on the rejuvenating agent type and amount. Ultimately, the aging resistance of rejuvenated reclaimed binders is equivalent to or better than that of B35 bitumen, unless higher rates of rejuvenating agents are used.

Therefore, this study found that the aging resistance of rejuvenated binders was comparable to that of the control bitumen, with less than a 5% difference in aging ratios. This result is consistent with the findings of Ma et al. [17], who reported that the direct addition of rejuvenators to reclaimed bitumen yielded better aging resistance than other methods.

### 3.5. Rutting and Fatigue Performance Parameters of Bitumen Samples

**Figure 13:**
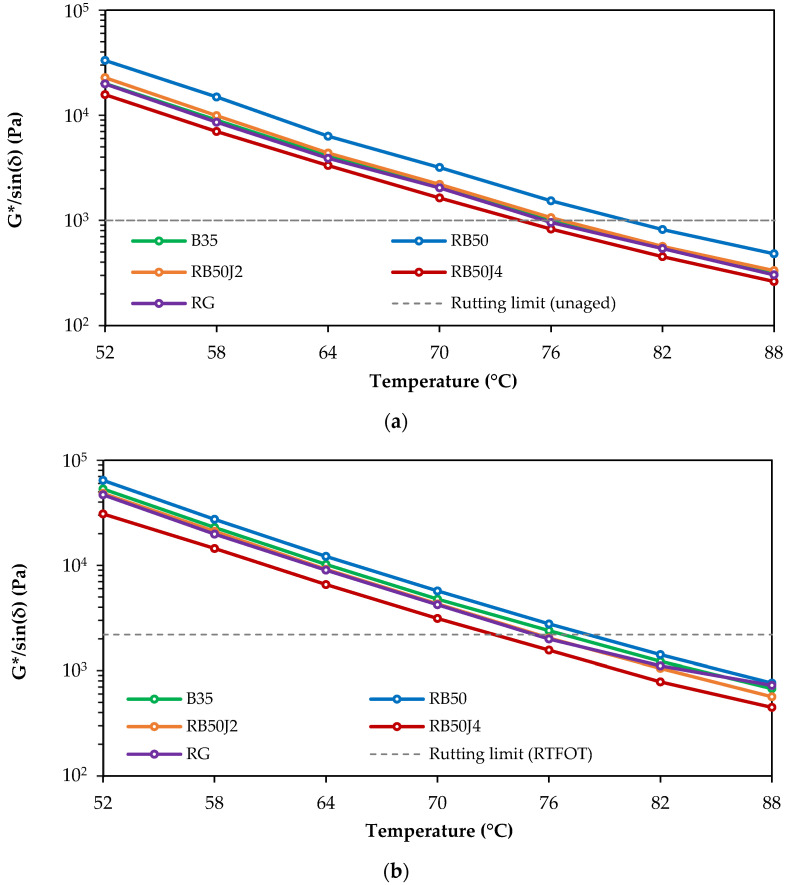
Rutting parameter variation in bitumen samples: (**a**) unaged binders; (**b**) RTFOT aged binders.

Figure 13 illustrates the variations in the G*/sin(δ) parameter with temperature for both unaged (Figure 13a) and short-term aged bitumen samples (Figure 13b).

As the temperature increases, the viscous behavior dominance causes the G* value to decrease and the δ value to increase, decreasing the G*/sin(δ) parameter. Figure 13 shows that the RB50 sample exhibits the highest rutting resistance, while the RB50J4 sample exhibits the lowest. Meanwhile, the RB50J2, RG, and B35 samples show similar performance. The temperature difference (approximately 5 °C) in controlling the rutting limit between the RB50 and RB50J4 samples highlights the rejuvenating agent’s significant effect in recovering the reclaimed bitumen’s maltene phase, making the bitumen softer and more flexible for pavement use. However, it also represents a reduction in the rutting resistance of RB50J4 samples.

**Figure 14:**
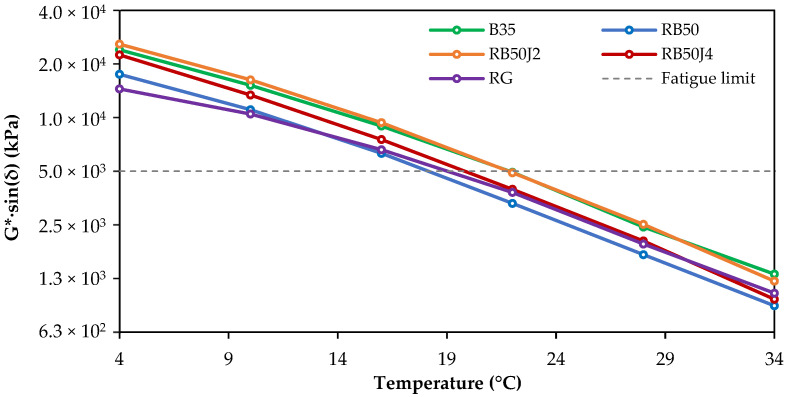
Fatigue parameter variation in PAV-aged binders.

The rheological characteristics of asphalt binders aged by the PAV method, associated with their fatigue resistance, were evaluated at intermediate temperatures (0 to 40 °C) through changes in the G*·sin(δ) parameter, as shown in Figure 14.

The results show that the RG and RB50J4 bitumen samples perform better against fatigue, confirming previous observations at high frequencies in the stiffness master curves. Surprisingly, RB50 bitumen (without rejuvenator) was the best-performing binder in this parameter, possibly because of the lower aging ratio of this binder after PAV conditioning. These results confirm the behavior of the bitumen samples shown at high frequencies in the master curves of long-term aged binders (Figure 9b), where these bitumen samples presented a behavior softer than B35 and RB50J2, because of their higher phase angles and lower complex moduli.

### 3.6. Large Amplitude Rheological Properties at High Temperatures

The evaluation of the rheological properties of rejuvenated bitumen samples for large amplitude strains followed the standard procedure of the multiple stress creep recovery test by applying a significant stress level of 3200 kPa beyond the linear viscoelastic regime at 60 °C. This test was conducted for the control bitumen B35 and the rejuvenated bitumen samples (i.e., RB50J2, RB50J4, RG). The corresponding non-recovered deformation (J_nr_) and percentage recovery (%R) results are presented in Table 3.

The results show that the bitumen samples prepared with the rejuvenator additive (RB50J2 and RB50J4) have higher non-recovered deformation and lower percentage recovery, demonstrating lower rutting resistance under extreme loading conditions. This observation applies to the unaged and the short- and long-term aged bitumen samples, which implies that part of the maltene fraction incorporated by the rejuvenator helps to maintain the bitumen deformable even after the two aging conditioning processes. The effect of the rejuvenator amount on the rutting properties evaluated in this test is apparent considering the higher J_nr_ and lower %R values of the RB50J4 samples with 0.4% rejuvenator compared to the RB50J2 samples with 0.2% rejuvenator. Therefore, these results confirm other authors’ observations in Section 1 (Introduction) about controlling the rejuvenator quantity used in recycled mixtures to avoid rutting problems. For this study, the binder incorporating 0.4% rejuvenating additive presented non-recovered deformation values 2.0 and 1.5 times higher than the control bitumen (B35) for the unaged and RTFOT samples. Therefore, 0.4% can be defined as the maximum rejuvenator incorporation rate for recycled mixtures with 30% reclaimed asphalt to avoid rutting problems. Thus, rejuvenation rates between 0.2% and 0.4% should be selected depending on the in-service climate conditions, reducing the incorporation in hot climates to avoid rutting problems and increasing its use in mild or cold climates where improved flexibility against cracking is a more critical issue.

In contrast, the bitumen sample RG with the regenerator binder showed permanent deformation results in the MSCR test identical to those of the control bitumen (B35) before and after aging. Thus, this rejuvenated bitumen has better resistance to rutting. However, it is less adaptable for different recycling scenarios owing to the fixed percentage of the rejuvenator agent in the commercial bitumen.

At last, the increased percentage recovery (%R) of all binders observed after PAV aging confirms a more significant presence of asphaltenes (the group with molecular structures exhibiting elastic behavior) and a reduction in the percentage of maltenes (responsible for viscous behavior).

Thus, the MSCR test showed that binders with 0.4% rejuvenator had higher non-recovered deformation and lower percentage recovery, suggesting lower rutting resistance. This result is in line with those reported by other studies, which mentioned that excessive use of rejuvenators could lead to reduced rutting performance due to increased phase angles and decreased stiffness at higher temperatures [49,50,51,52,53]. However, the study also highlighted that appropriate rejuvenator quantities could balance flexibility and rutting resistance, a point also noted by Zhang et al. [1] and Sun et al. [40].

## 4. Conclusions

This study evaluated the feasibility of using 30% reclaimed asphalt on pavements while maintaining the rheological characteristics of the rejuvenated reclaimed binder comparable to pure 35/50 pen-grade bitumen. Particular attention was also given to the aging resistance of the rejuvenated binders. Several key findings emerged from this study:

- The RB50J4 sample, containing 0.4% rejuvenator, exhibited similar penetration grade, softening point, and viscosity to pure 35/50 bitumen, with reduced susceptibility to aging.
- A 30:70 blend of reclaimed binder to pure 50/70 bitumen without a rejuvenating agent failed to match the rheological properties of pure 35/50 bitumen.
- The type and amount of rejuvenator significantly influenced the rheological parameters. The RB50J4 sample showed a stiffness reduction of between 20% and 55% compared to the sample without a rejuvenator. Higher percentages of rejuvenators decreased the complex modulus and increased the phase angle, yielding softer bitumen.
- The rejuvenators effectively reduced the crossover temperature, with RB50J4 showing the lowest crossover temperature and enhanced aging resistance.
- The RB50J4 sample exhibited decreased rutting resistance compared to pure 35/50 bitumen. However, using 0.2% to 0.4% rejuvenator can achieve suitable rutting performance levels while maintaining flexibility.
- Properly controlling the rejuvenator quantity in recycled mixtures is essential for balancing stiffness, aging resistance, and rutting performance.

These findings underscore the potential of using reclaimed bitumen with specific rejuvenating agents to achieve pavement performance comparable to pure 35/50 pen-grade bitumen in recycled mixtures incorporating 30% reclaimed asphalt.

Recommendations for future developments include validating the current study through performance testing on recycled asphalt mixtures with 30% RA to verify the bitumen performance results and further study fracture and cracking resistance. Recommendations for field applications include selecting appropriate rejuvenator types and quantities based on environmental and traffic conditions to optimize performance.

## Figures and Tables

**Table 1:** Particle size distribution of RA material.

Sieve Size (mm)	Percentage Passing (%)	
	Sample without Bitumen	Sample with Bitumen
31.5	100	100
20	99	91
14	98	83
10	93	73
4	66	43
2	49	29
0.5	25	12
0.125	11	4
0.063	6	3

**Table 2:** Properties of the rejuvenator used in this research.

Typical Properties	Unit	Value
Appearance	-	liquid
Color	-	brown
Viscosity at 25 °C	mPa·s	50–150
Density at 20 °C	g/cm^3^	0.85–0.95
Flash point	°C	200

**Table 3:** Results of the multiple stress creep recovery tests for a stress level of 3200 Pa and a temperature of 60 °C for the unaged and short- and long-term aged bitumen samples.

Bitumen Sample	Unaged		RTFOT		PAV	
	J_nr_ (kPa^−1^)	%R	J_nr_ (kPa^−1^)	%R	J_nr_ (kPa^−1^)	%R
B35	1.38	2.5%	0.43	11.8%	0.08	33.1%
RB50J2	1.85	1.2%	0.43	11.0%	0.24	22.8%
RB50J4	2.74	0.2%	0.64	7.3%	0.33	16.4%
RG	1.42	2.7%	0.45	11.7%	0.09	33.6%

## Data Availability

The original contributions presented in the study are included in the article, further inquiries can be directed to the corresponding author.
